# Sedation practice in the intensive care unit: a UK national survey

**DOI:** 10.1186/cc7141

**Published:** 2008-12-01

**Authors:** Henrik Reschreiter, Matt Maiden, Atul Kapila

**Affiliations:** 1 Royal Adelaide Hospital, North Terrace, Adelaide, SA 5000, Australia; 2 Royal Berkshire and Battle Hospital NHS Trust, London Road, RG1 5AN, Reading, UK

## Abstract

**Introduction:**

The purpose of this study was to evaluate sedation practice in UK intensive care units (ICUs), particularly the implementation of daily sedation holding, written sedation guidelines, sedation scoring tools and choice of agents.

**Methods:**

A national postal survey was conducted in all UK ICUs.

**Results:**

A total of 192 responses out of 302 addressed units were received (63.5%). Of the responding ICUs, 88% used a sedation scoring tool, most frequently the Ramsey Sedation Scale score (66.4%). The majority of units have a written sedation guideline (80%), and 78% state that daily sedation holding is practiced. A wide variety of sedating agents is used, with the choice of agent largely determined by the duration of action rather than cost. The most frequently used agents were propofol and alfentanil for short-term sedation; propofol, midazolam and morphine for longer sedation; and propofol for weaning purposes.

**Conclusions:**

Most UK ICUs use a sedation guideline and sedation scoring tool. The concept of sedation holding has been implemented in the majority of units, and most ICUs have a written sedation guideline.

## Introduction

Patients requiring mechanical ventilation in the intensive care unit (ICU) usually require a sedating agent [1]. Sedation reduces the negative physiological effects of the stress response to mechanical ventilation [2,3] and may reduce the psychological issues patients may face after critical illness [4]. However, excessive sedation may be harmful. Over-sedation can contribute to hypotension, venous thrombosis, prolonged ventilation, an increased risk for pneumonia and a prolonged stay in the ICU, with an increasing burden on staff, bed availability and associated costs [5,6].

Recent evidence indicates that the choice of sedating agents, frequency of administration and regular assessment of sedation contribute to patient outcomes [7-9]. Kress and coworkers [7], in 2000, demonstrated that daily interruption of sedation reduced ventilation duration, ICU length of stay, complications such as venous thromboembolic disease, upper gastrointestinal bleeding and bacteraemia, and the incidence of post-traumatic stress disorder [10,11].

There have been a number of systematic reviews of sedation practice in the ICU and subsequent evidence-based clinical practice guidelines for sedation [12-16]. However, uptake of these evidence-based guidelines is variable. Sedation surveys in a range of countries have demonstrated different practices in the management of sedation [17-21]. The last survey of sedation practice in UK ICUs was published in 2000 [22], before the concept of daily sedation holding was published. The rate of implementation of current sedation guidelines in UK ICUs is unknown.

This UK national survey was performed to assess the impact of published trials and guidelines on ICU sedation practice since 2000.

## Materials and methods

A tick-box questionnaire was developed to survey sedation practice [see Additional data file 1]. The questionnaire was sent to all UK ICUs. The list of units was obtained from the Intensive Care National Audit & Research Centre and cross-referenced with the Directory of Critical Care 2006 (CMA Medical Data, Loughborough). The questionnaire and covering letter was addressed to the 'Clinical Director' of the ICU, and a stamped self-addressed return envelope was provided.

The local ethics committee (Royal Berkshire NHS Foundation Trust, Reading, UK) was approached, but formal processing and approval was deemed unnecessary.

The questionnaire was posted out in November 2006. Those ICUs that did not reply received follow-up questionnaires in December 2006 and March 2007. The last response was received in June 2007.

The data were entered into a database (Microsoft Excel Office 2003; Microsoft Corp., Redmond, WA, USA). The data were then read into version 9.1 of the SAS^®1 ^system (SAS Institute Inc., Cary, NC, USA) running under Microsoft Windows XP, where they were summarized and analyzed. Data were cross-tabulated as appropriate and Mantel-Haenszel χ^2 ^tests were used for analysis. Paired *t*-tests were used to look for any differences for cost versus duration of action. Differences were deemed to be statistically significant at *P *< 0.05.

## Results

A total of 302 UK ICUs were identified and responses were received from 192 (63.5%). Seven of these responses were excluded from further analysis; five were high dependency units that do not admit ventilated patients, and two questionnaires were returned blank. The denominator used for the results and statistical analysis was 185. The geographical distribution revealed that 155 hospitals were situated in England, 15 in Scotland, 10 in Wales and five in Northern Ireland. The demographic data of the replying ICUs are outlined in Table 1 and reveal that a wide range of ICUs were surveyed.

**Table 1:** General Data

Variable	Number of units (%)
Number of beds	

0 to 4	26 (14%)
5 to 8	99 (53%)
9 to 12	36 (19%)
>12	24 (13%)
	Total: 185 units

Number of ICU admissions/year^a^	

<250	17 (9%)
250 to 500	83 (45%)
500 to 750	43 (23%)
750 to 1,000	22 (12%)
>1,000	17 (9%)
	Total: 182 units

% ventilated patients^a^	

0 to 25	7 (4%)
26 to 50	45 (24%)
51 to 75	79 (43%)
76 to 100	49 (26%)
	Total: 180 units

Type of patients^b^	

Surgical	157 (85%)
Medical	156 (84%)
Cardiac	13 (7%)
Neurological	21 (11%)

^a^No answer to the question was given by some units. ^b^More than one type of patient in an ICU was possible. ICU, intensive care unit

Table 2 illustrates that 88.1% of UK ICUs utilize a sedation scoring tool. The Ramsey Sedation Scale score [23] is the most widely used (66.5%). A number of ICUs have developed their own sedation scores (details unknown), and have named them after their place of development/workplace.

**Table 2:** Sedation scoring practice

Question	Number of units (%)
Do you use a sedation score?	
Yes	163 (88.1%)
No	19

Which sedation score do you use? (several answers possible)	
Ramsey Sedation Scale	123 (66.5%)
Richmond Agitation Sedation scale	10 (5.4%)
Bispectral Index	4 (2.1%)
Score of the UK Intensive Care Society	7 (3.8%)
Sheffield	4 (2.2%)
Bloomsbury	2 (1.1%)
Cambridge	2 (1.1%)
Cook	3 (1.6%)
Local scoring system, unspecified	3 (1.6%)
Other, but not specified	7 (3.8%)
No answer given	3 (1.6%)

Do you have a sedation guideline?	
Yes	148 (80%)
No	37

Do you practice daily sedation holding?	
Yes	144 (77.8%)
No	41

Do you audit your compliance with your sedation holding guideline?	
Yes	99 (53.5%)
No	82

If you audit your compliance with your sedation holding practice, what is your compliance?	
0% to 40%	5
40% to 60%	14
60% to 80%	26
80% to 90%	23
90% to 100%	24

The sum of answers is less than 185 in some cases as no answer to the question was given by some units.

Most UK ICUs (80%) have a written sedation guideline and 78% practice daily sedation holding. However, only 53% of ICUs audit compliance with their guidelines (Table 2). No difference could be observed between units of different size or depending on number of admissions (Table 3).

**Table 3:** Size of unit and sedation practice

Number of beds	Number of ICUs (n = 185)	Sedation guideline (n = 148)^#^	No guideline (n = 37)	Daily sedation holding (n = 144)	No sedation holding (n = 41)
0 to 4	26	19 (73%)	7 (27%)	20 (77%)	6 (23%)
5 to 8	99	78 (79%)	21 (21%)	78 (79%)	21 (21%)
9 to 12	36	30 (83%)	6 (17%)	31 (86%)	5 (14%)
>12	24	20 (83%)	3 (13%)	15 (63%)	9 (37%)

Percentages are out of the ICUs of that size group. No statistically significant differences were observed. # One ICU with more than 12 beds left this question unanswered. ICU, intensive care unit.

Neuromuscular blocking agents are infrequently used, with 71% of ICUs using it less than 5% of the time. However, 7% of ICUs use muscular blocking agents in more than 10% of their patients; these ICUs were predominantly neurosurgical (Table 4).

**Table 4:** Neuromuscular blocking agents used

% of patients	Number of ICUs	Number of neurological ICUs
0% to 5%	128 (71%)	4 neurological ICUs
6% to 10%	39 (22%)	7 neurological ICUs
11% to 15%	6 (3%)	2 neurological ICUs
16% to 20%	6 (3%)	5 neurological ICUs
>20%	2 (1%)	1 neurological ICU, one cardiac-ICU

The denominator was 181 (four responding ICUs did not answer this question). ICU, intensive care unit.

**Figure 1:**
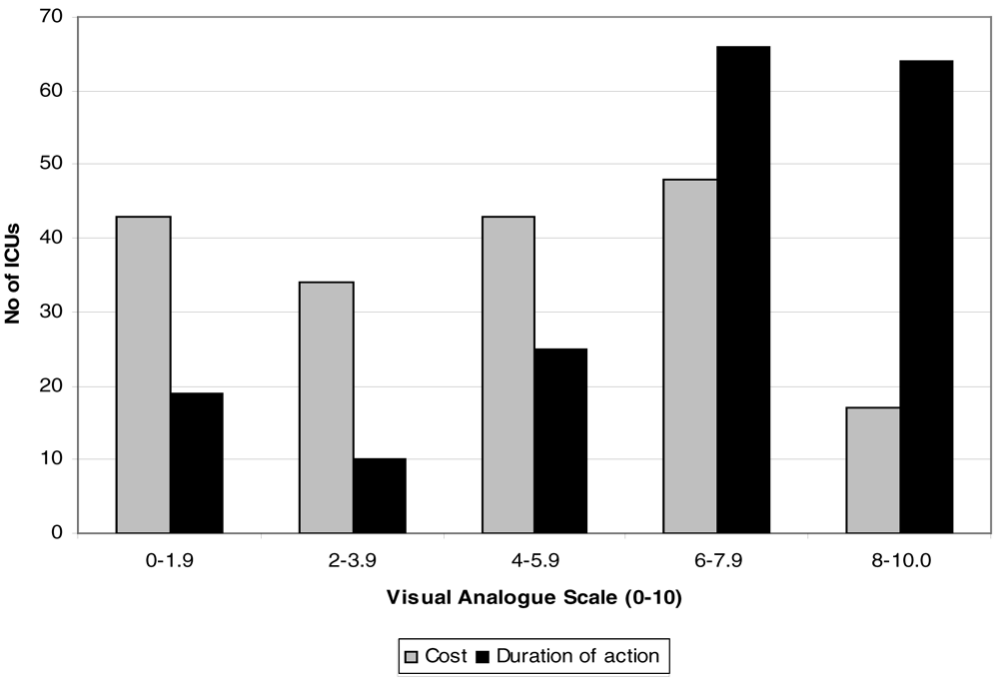
**Importance of cost and duration of action on choice of agent**. A total of 185 units are included in this analysis. VAS, visual analogue scale (range: 0 = not important to 10 = most important).

According to visual-analogue scale assessment (0 = not affecting decision and 10 = main factor), choice of sedating agent is strongly influenced by duration of action. In comparison, cost of the sedating drug has less of an influence on sedation choice (mean visual-analogue scale score cost 4.4 versus duration of action 6.4; *P *< 0.0001; Figure 1).

A range of sedating agents are used by the surveyed ICUs (Table 5). Propofol is the most frequently used sedating agent for patients with expected duration of ICU admission less than 24 hours. For an expected ICU admission of more than 24 hours, midazolam and propofol are the most commonly used agents. During ventilator weaning, propofol is used most frequently, with clonidine being the next most commonly used agent.

**Table 5:** Agents used stratified by expected length of stay in the ICU

Expected length of stay in the ICU	<24 hours	>24 hours	Weaning
For sedation			
Propofol	181	111	65
Midazolam	23	136	8
Clonidine	2	20	22
Lorazepam	2	13	3
Haloperidol	1	5	7
Diazepam	1	2	1

For analgesia			
Morphine	51	125	14
Fentanyl	45	46	16
Alfentanil	96	67	24
Remifentanil	43	11	24
Ketamine	1	3	0

Multiple answers were possible. Values are numbers of units. A total of 185 units are included in this analysis. ICU, intensive care unit.

A wider range of analgesic agents is used during the sedation of ICU patients. For short-term analgesia, alfentanil, morphine, fentanyl and remifentanil are commonly used. For longer expected sedation (>24 hours), morphine is the most commonly used agent.

## Discussion

### Sedation scoring

The majority of responding ICUs (88%) in our survey use a sedation scoring system. This has increased considerably since the UK survey in 2000, when 67% of hospitals used a scoring system [22]. Despite this increased uptake since the last survey and favourable comparison with other countries, an evidence-based approach is still not universally followed.

Numerous sedation assessment tools have been developed to minimize this risk for over-sedation. Some sedation scales have been validated against other scales in patients (for example, Riker Sedation-Agitation Scale [24,25], Motor Activity Assessment Scale [26], Vancouver Interaction and Calmness Scale [27], and more recently the Richmond Agitation-Sedation Scale [28,29], published after the American College of Critical Care Medicine (ACCM) guidelines [12]. The latter has shown an excellent performance, not only with regard to inter-rater reliability and validity, but it is also the first score to detect changes over time in the critically ill patient. However, no consensus yet exists in an international guideline regarding which assessment tool to use, and validation itself is problematic because of the lack of a standard to validate against that is not based on opinion.

The uptake of the Richmond Agitation-Sedation Scale has been slow in previous surveys. It did not feature in the 2005 German survey [17] or in the Canadian survey conducted in 2006 [18], with one unit reporting its use in the 2007 German update survey. The French survey in 2007 [21] and our study are the first reports of its use being more widespread.

The sedation scale used most commonly in this survey was the Ramsey Sedation Scale [23], with 66.5% of ICUs using this sedation assessment. Furthermore, most of the other scales used are adaptations of the Ramsay Sedation Scale (for example, the UK Intensive Care Society sedation scale). The choice of sedation scoring tool has not changed since the last UK survey, with the Ramsay Sedation Scale score being used most commonly then (40 out of 142 units stating that they used a sedation scoring system in 2000) [22].

The widespread use of the Ramsay Sedation Scale is in contrast to the ACCM guidelines [12], which recommend use of the validated assessment scores, such as the Motor Activity Assessment Scale, Riker Sedation-Agitation Scale, and Vancouver Interaction and Calmness Scale. Its advantages appear to be familiarity to staff and simplicity, and it is the scale that has been most commonly used historically despite its clinical limitations. However, the Ramsay Sedation Scale lacks clear discrimination and exhibits considerable inter-rater variability [30].

The practice of sedation assessment in the UK differs from that in other countries. In Germany in 2005 only 51% of responding ICUs report sedation monitoring, with the Ramsay Sedation Scale used 'almost exclusively'[17], and in Canada in 2006 only 49% of responding ICUs utilized sedation monitoring, with 69% of ICUs using the Ramsay Sedation Scale [17,18,20]. Our results are consistent with a 2001 sedation survey conducted in European ICUs [28], which showed that ICUs in the UK use sedation scales more frequently than do those in all other participating European countries (72% of UK ICUs).

### Sedation guidelines

It has been shown in many but not all studies over the past decade that an ICU sedation protocol results in fewer days on the ventilator, a shorter stay in the ICU and reduced costs [31-37]. Despite the mostly good evidence and a comparably easy and cheap means of improving care, not all hospitals have implemented a formal sedation guideline.

In this survey, 80% of the responding hospitals have an operating sedation guideline, which has increased sharply since 2000, when 43% of participating hospitals stated that they had a written guideline. A German survey conducted in 2007 [20] revealed that 46% of hospitals used a sedation guideline, and the Canadian survey in 2006 [18] reported that 29% of ICUs used a sedation protocol.

Our high rate of ICUs reporting use of a sedation guideline could reflect reporter bias, because units with more interest in sedation may be more likely to respond to this questionnaire. However, there has been increased utilization of sedation guidelines in Germany in the recent years, suggesting that our results may reflect actual change in practice [20].

### Sedation holding

In 2000, Kress and coworkers [7,11] showed that daily withholding of sedative agents led to reduced length of ICU stay, less ventilator time, fewer ICU complications and fewer neurological investigations. Subsequent studies by the same group demonstrated daily sedation withholding to be safe in patients with ischaemic heart disease, and that it reduces the psychological sequelae of critical illness [10,38]. A different group, however, raised safety concerns in a trial including a high percentage of patients (around 30% to 40%) with alcohol and other drug use disorders, emphasizing that patient selection and an individualized approach is important [39].

A recent pilot trial addressed the issue of safety and feasibility of daily interruption of sedation with simultaneous use of protocolized sedation [40]. The authors concluded that in their pilot daily trial sedation practice was not associated with an increased incidence of adverse events.

Sedation withholding is now part of the 'ventilator care bundle', as outlined by the UK Department of Health [33], and recommended by the Surviving Sepsis Campaign [41].

In our survey, 78% of the ICUs state that they practice daily sedation holding. By comparison, the reported proportion of ICUs practicing sedation withholding in Canada is 40%, in Denmark it is 31% and in Germany it is 34% [18-20]. However, our study revealed that only 53% of ICUs audit their use of daily sedation holding, which suggests that the number of ICUs practicing sedation holding effectively is probably lower than the 78% stated.

There may be many reasons why ICUs are not practicing daily sedation holding. Devlin and coworkers [42] surveyed American clinicians in 2004 and found that some ICUs were not adopting sedation holding because of a lack of nursing acceptance of this practice, a potential increase in patient self-harm, potential for respiratory compromise and concern about patient comfort.

The high rates of sedation holding reported in this study may reflect increasing awareness and acceptance of the technique and most subsequent studies supporting its safety and benefit.

### Choice of agents used

The sedation guideline published by the ACCM [12] recommends use of fentanyl or morphine for analgesia, midazolam or propofol for short-term sedation, and lorazepam for longer term sedation. The practice in the UK differs greatly from these guidelines. Alfentanil is used more commonly than fentanyl or morphine, likely because of its lesser degree of accumulation and shorter duration of action. For patients expected to require sedation for longer than 24 hours, morphine and midazolam were most frequently chosen, whereas lorazepam is rarely chosen.

Our survey illustrated that the duration of action of the sedating agent was a more important factor in choice of sedating agent than its cost. This concurs with the German sedation survey [20]. It will be interesting to observe whether newer short-acting but more expensive agents (for instance, remifentanil and dexmedetomidine) are chosen for sedation in the future.

### Neuromuscular blocking agents

Muscle relaxing agents are infrequently used in UK ICUs. The few ICUs using neuromuscular blocking agents (NMBAs) in more than 10% of patients were mostly neurological ICUs. The infrequent use of NMBAs may reflect the increasing emphasis on lighter levels of sedation and the concerns regarding critical illness neuropathy and myopathy [43].

### Study limitations

This study shares the limitations of all surveys in that reporter bias cannot be excluded. Furthermore, only the head of the department was addressed; the answers may therefore only reflect the individual's practice, and may not be representative for the entire unit. Past surveys, however, would have faced similar limitations, and given our good response rate, comparison with surveys in the past and in other countries can be made. The wide range of units responding make it likely that our results reflect actual UK practice appropriately, within the constraints of self-reporting practice.

## Conclusion

An increasing number of ICUs in the UK utilize a sedation guideline and a sedation scoring tool. The Ramsey Sedation Scale is the most frequently chosen assessment score. Sedation holding is done by most but not all of the ICUs. Its implementation compares favourable with that identified in other international sedation surveys. The choice of sedating agent is quite variable and differs from that in other countries. Choice of sedating agent is directed more by duration of action rather by cost. NMBAs are infrequently used outside neurological ICUs.

## Key messages

• The majority of units have a standardized approach to sedation management, using a sedation guideline, sedation scoring and daily sedation holding.

• In contrast to published guidelines and existing evidence, there is still a considerable number of ICUs that do not practice effective daily sedation holding.

• Wide variation exists in the choice of sedating or analgesic agent, with the short-acting opioid alfentanil being a popular choice.

• Only a minority of ICUs use NMBAs regularly.

• Choice of sedating agent is directed more by duration of action rather by cost.

## Abbreviations

ACCM: American College of Critical Care Medicine; NMBA: neuromuscular blocking agent; ICU: intensive care unit.

## Competing interests

The authors declare that they have no competing interests.

## Authors' contributions

HR and AK made substantial contributions to the conception, design, analysis and interpretation of the data. MM had substantial involvement in revising and drafting the article, and in interpreting the data. All authors contributed to drafting and revising the article, and approved the final manuscript.

## Supplementary Material

Additional file 1 A Word document containing the questionnaire sent to all UK ICUs. Click here for file
