# Supplementary material for: Sedation practice in the intensive care unit: a UK national survey
Source: Crit Care. 2008 Dec 1;12(6):R152. doi: 10.1186/cc7141 (PMC2646317; doi:10.1186/cc7141)
Supplement: Additional file 1 — A Word document containing the questionnaire sent to all UK ICUs. [file cc7141-S1.doc]

**Questionnaire**

1. Number of regularly staffed Level 3 (ICU) beds:

| 0 - 4 | 5 -8 | 9 -12 | >12 |
| --- | --- | --- | --- |

1. Total admissions to your unit per year:

| <250 | 251 - 500 | 501 - 750 | 751 – 1000 | >1000 |
| --- | --- | --- | --- | --- |

1. Percentage of admissions ventilated:

| 0 – 25% | 26 – 50% | 51 – 75% | 76 – 100% |
| --- | --- | --- | --- |

1. Predominant origin of patients admitted (several answers possible):

| surgical | medical | cardiac | neuro | paediatric |
| --- | --- | --- | --- | --- |

**Sedation questionnaire**

1. If you do use a sedation scale, which one:
   1. (mod) Ramsay score ⁭
   2. RASS ⁭
   3. Bispectral Index Score (BIS) ⁭
   4. Other ⁭ _____________________
   5. We do not use a sedation scale. ⁭
2. Do you have a written sedation guideline: ⁭ Yes ⁭ No
3. Do you practice daily sedation holding? ⁭ Yes ⁭ No
4. Do you audit your sedation and sedation holding

practice regularly: ⁭ Yes ⁭ No

1. If you do audit your sedation and sedation holding practise, what is approximately your compliance with you guidelines:

| Not audited | 0 – 40% | 41 -60% | 61 – 80% | 81 – 90% | 91 – 100% |
| --- | --- | --- | --- | --- | --- |

1. In how many of your patients approximately do you use Neuro-muscular blocking agents (NMBAs):

| 0 – 5% | 6 – 10% | 11 – 15% | 16 – 20% | >20% |
| --- | --- | --- | --- | --- |

1. How much does cost play a role in your choice of agents for sedation/analgesia (please mark with a vertical line on the 10 cm Visual analogue scale (VAS))?

Not at all Main factor

1. How much does the expected duration of sedative / analgesic infusions used influence the choice in each patient (i.e.: “short” vs. “long” acting agents)?

Not at all Main factor

1. For patients expected to stay <24 hrs, which agent or technique do you regularly use for continuous sedation/analgesia (tick one or more)?

| *Sedation:*  Midazolam  Propofol  Lorazepam  Diazepam  Haloperidol  Clonidine  Other:_________ | ⁭  ⁭  ⁭  ⁭  ⁭ | *Analgesia:*  Morphine  Fentanyl  Alfentanil  Remifentanil  Ketamine  Other:_________ | ⁭  ⁭  ⁭  ⁭  ⁭  ⁭ | *Misc.*  Epidural  PCEA  Periph. block  PCA  Other:________ | ⁭  ⁭  ⁭  ⁭  ⁭ |
| --- | --- | --- | --- | --- | --- |

1. For patients expected to stay >24 hrs, which agent or technique do you regularly use for continuous sedation/analgesia (tick one or more)?

| *Sedation:*  Midazolam  Propofol  Lorazepam  Diazepam  Haloperidol  Clonidine  Other:_________ | ⁭  ⁭  ⁭  ⁭  ⁭  ⁭  ⁭ | *Analgesia:*  Morphine  Fentanyl  Alfentanil  Remifentanil  Ketamine  Other:_________ | ⁭  ⁭  ⁭  ⁭  ⁭  ⁭ | *Misc.*  Epidural  PCEA  Periph. block  PCA  Other:________ | ⁭  ⁭  ⁭  ⁭  ⁭ |
| --- | --- | --- | --- | --- | --- |

1. Do you change your sedative drugs used for weaning patients off ventilatory support?

⁭ Yes ⁭ No

1. If yes, for patients weaning from ventilatory support, which agent or technique do you regularly use for continuous sedation/analgesia (tick one or more)?

| *Sedation:*  Midazolam  Propofol  Lorazepam  Diazepam  Haloperidol  Clonidine  Other:_________ | ⁭  ⁭  ⁭  ⁭  ⁭  ⁭  ⁭ | *Analgesia:*  Morphine  Fentanyl  Alfentanil  Remifentanil  Ketamine  Other:_________ | ⁭  ⁭  ⁭  ⁭  ⁭  ⁭ | *Misc.*  Epidural  PCEA  Periph. block  PCA  Other:________ | ⁭  ⁭  ⁭  ⁭  ⁭ |
| --- | --- | --- | --- | --- | --- |
